# Supplementary material for: HS1BP3 negatively regulates autophagy by modulation of phosphatidic acid levels
Source: Nat Commun. 2016 Dec 22;7:13889. doi: 10.1038/ncomms13889 (PMC5412012; doi:10.1038/ncomms13889)
Supplement: Supplementary Information — Supplementary Figures, Supplementary Tables, and Supplementary References [file ncomms13889-s1.pdf]

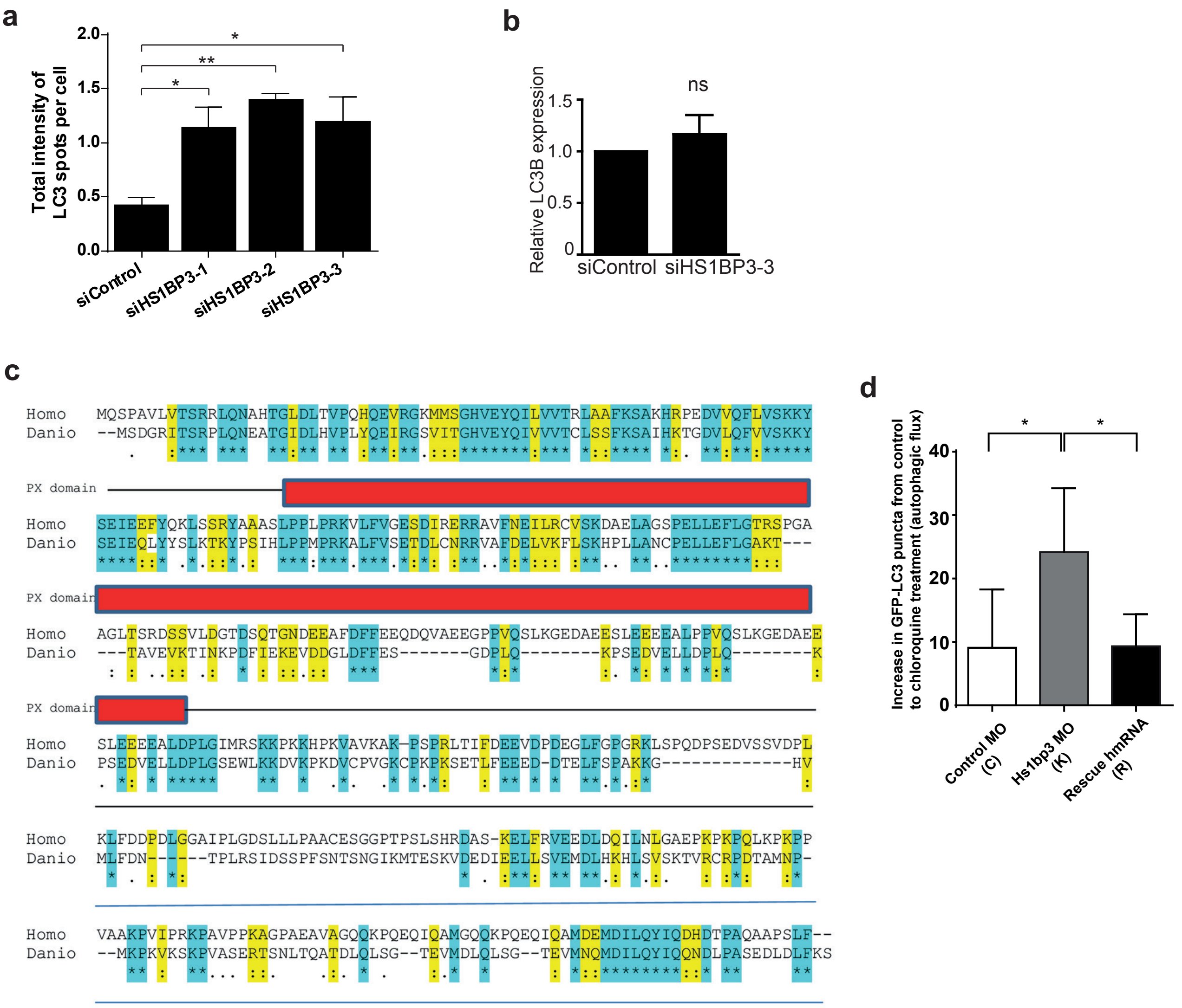

Supplementary Figure 1: HS1BP3 depletion increases autophagy in human cells and zebrafish

(**a**) HEK cells were transfected with siRNA against HS1BP3 and starved for 2 h before fixation, immunostaining against LC3 and imaging. The total intensity of endogenous LC3 spots per cell was quantified (mean +/- S.E.M., n = 3). 600 cells analyzed per condition. (**b**) LC3B mRNA levels were quantified by qPCR from siHS1BP3 or control transfected cells (mean +/- S.E.M., n = 3). (**c**) ClustalW alignment of Hs1bp3 amino acid sequences of human and Danio rerio. Identical and similar residues are boxed in cyan and yellow, respectively. The position of the conserved PX domain is indicated by a red box below the sequences. (**d**) Quantification of the difference in GFP-LC3 puncta count between the chloroquine treated and the untreated in embryos injected with control morpholino, Hs1bp3 translational blocking morpholino or Hs1bp3 translational blocking morpholino together with human Hs1bp3 mRNA (mean ± SEM, n = 3) . *: $p < 0.05$, **: $p < 0.01$, ns: non-significant.

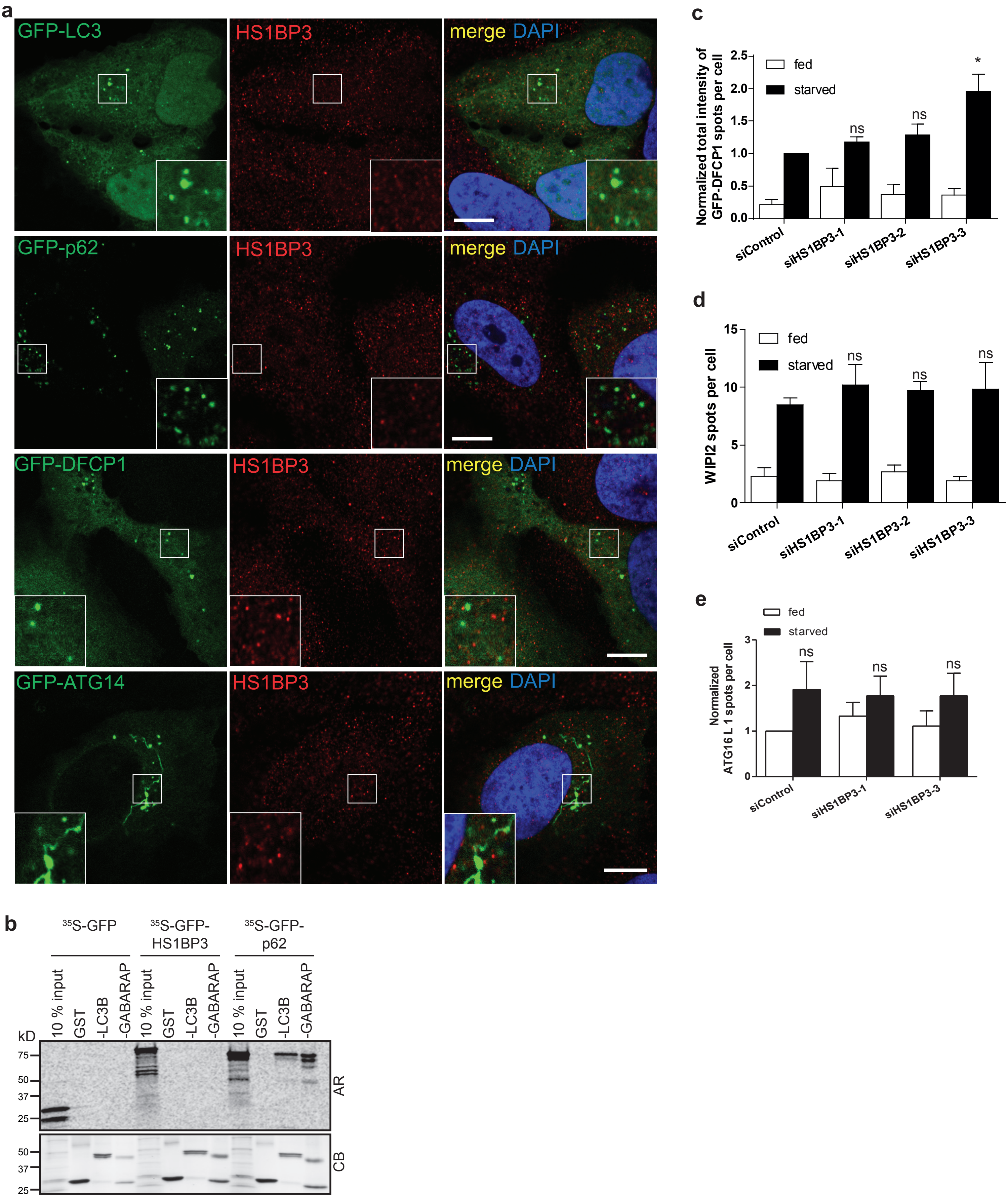

Supplementary Figure 2: Localization of endogenous HS1BP3 and its effects on early phagophore markers.
(**a**) HEK cells were transfected with the indicated GFP-tagged autophagy markers, starved, fixed and co-stained for endogenous HS1BP3. Scale bars 10 μm. (**b**) In vitro translated GFP, GFP-HS1BP3 or GFP-p62 was incubated with recombinant GST-tagged LC3B or GABARAP. Following GST pulldown, bound proteins were detected by autoradiography (AR) and GST proteins by Coomassie blue staining (CB). (**c**) HEK GFP-DFCP1 cells were transfected with siRNA against HS1BP3 and starved or not for 50 min before fixation and imaging. The total intensity of GFP-DFCP1 spots per cell was quantified and normalized to that of starved siControl cells (mean +/- S.E.M., n = 3). 1500 cells were analyzed per condition. (**d**) HEK GFP-DFCP1 cells were treated as in c, stained for endogenous WIPI2 and the number of WIPI2 spots per cell was quantified (mean +/- S.E.M., n = 3). (**e**) HeLa cells were transfected with either control or HS1BP3 siRNA, starved or not for 2 h before fixation and staining for endogenous ATG16L1. The number of ATG16L1 spots per cell was quantified (mean ± SEM, n = 3) *: p< 0.05, ns: non-significant.

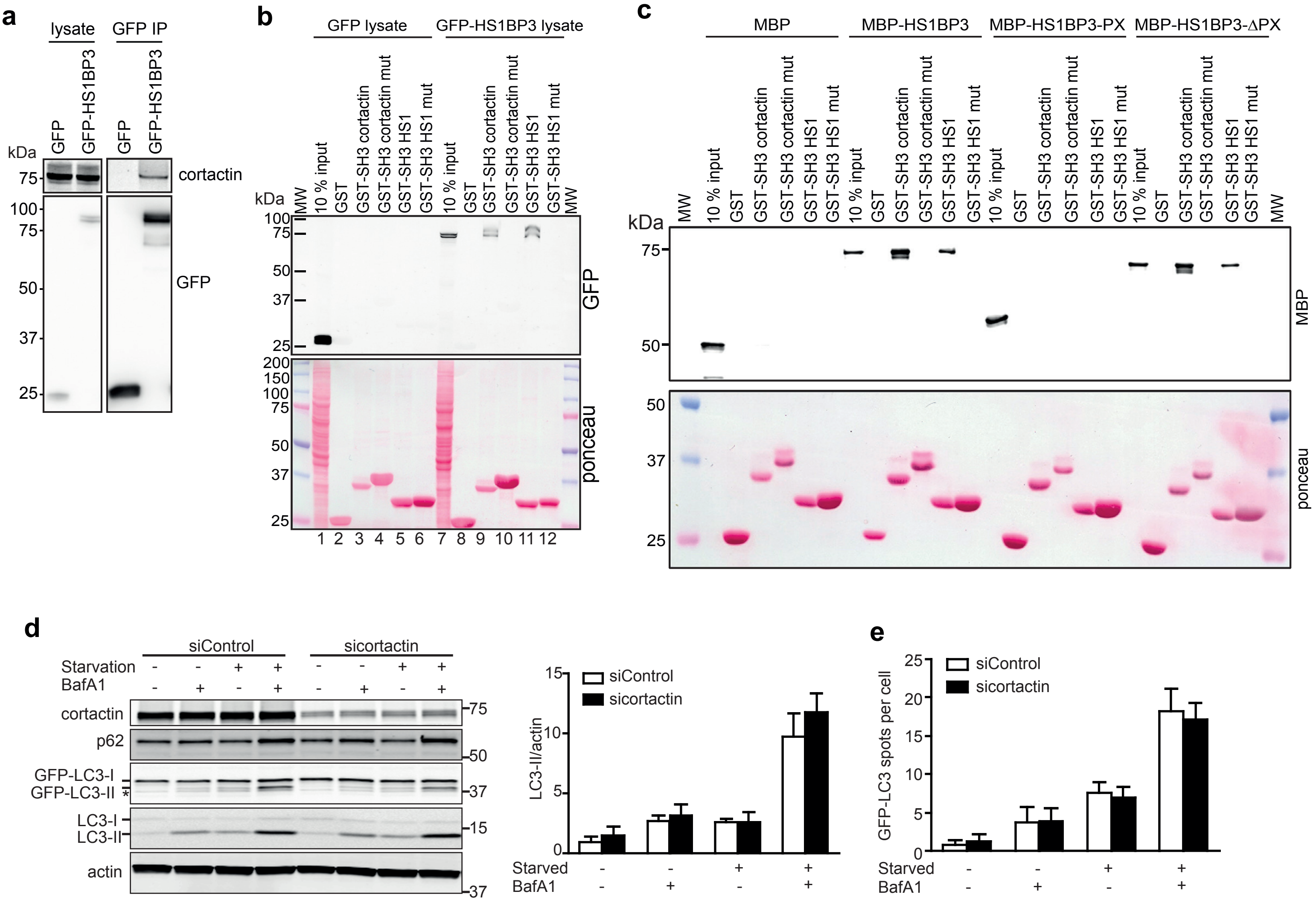

Supplementary Figure 3: HS1BP3 interacts with cortactin independently of its role in autophagy

(**a**) HeLa cell stably expressing tet-on GFP or GFP-HS1BP3 were induced by 50 ng/mL tetracycline for 16 h followed by immunoprecipitation using GFP trap. The resulting immunoprecipitates were separated by SDS-PAGE and analyzed by immunoblotting as indicated. (**b**) Lysates from HeLa GFP or GFP-HS1BP3 cells induced by 50 ng/mL tetracycline for 16 h were incubated with recombinant GST-tagged wild-type or mutated SH3 domain of cortactin or HS1, followed by pull-down using glutathione sepharose, separation by SDS-PAGE and immunoblotting against GFP. The membrane was stained with Ponceau S to visualize the GST-proteins and input lysate. (**c**) Recombinant MBP-tagged HS1BP3 full length or deletion mutants (HS1BP3-PX or HS1BP3-ΔPX) were incubated with recombinant GST-tagged wild-type or mutated SH3 domain of cortactin or HS1 followed by pull-down using glutathione beads, separation by SDS-PAGE and immunoblotting or Ponceau S staining as indicated. (**d**) HEK GFP-LC3 cells were transfected with siRNA against cortactin. 72 h later the cells were starved or not for 2 h in EBSS in the presence or absence of BafA1. Cell lysates were separated by SDS-PAGE and immunoblotted with the indicated antibodies. LC3-II/actin was quantified from immunoblots (mean +/- *S.E.M., n = 3)*. (**e**) HEK GFP-LC3 cells treated as in d were fixed and analyzed by fluorescent microscopy and high-content image analysis. The number of GFP-LC3 spots per cell was quantified (mean +/- S.E.M., n = 3). Around 1500 cells were quantified per condition. Scale bars are 10 µm.

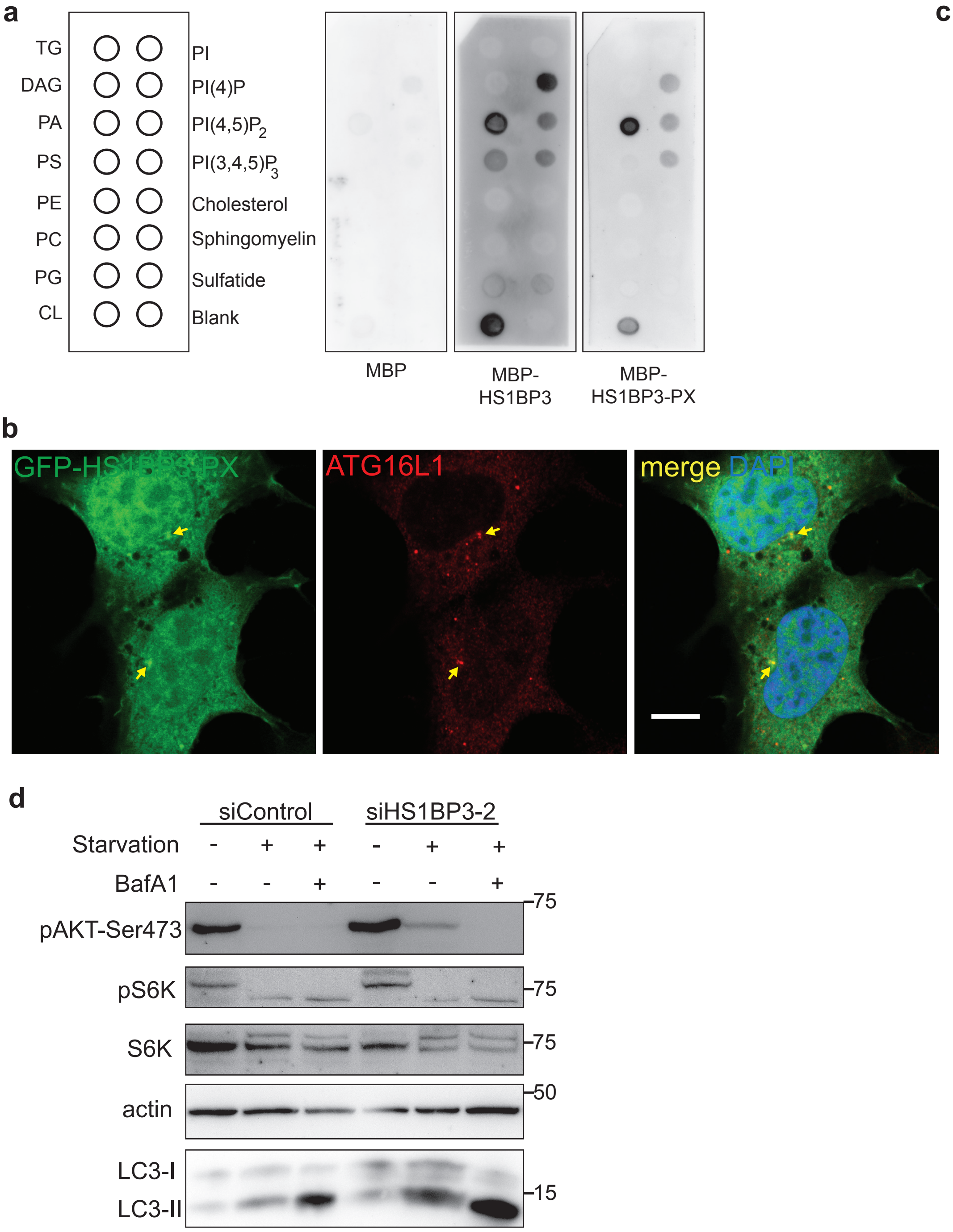

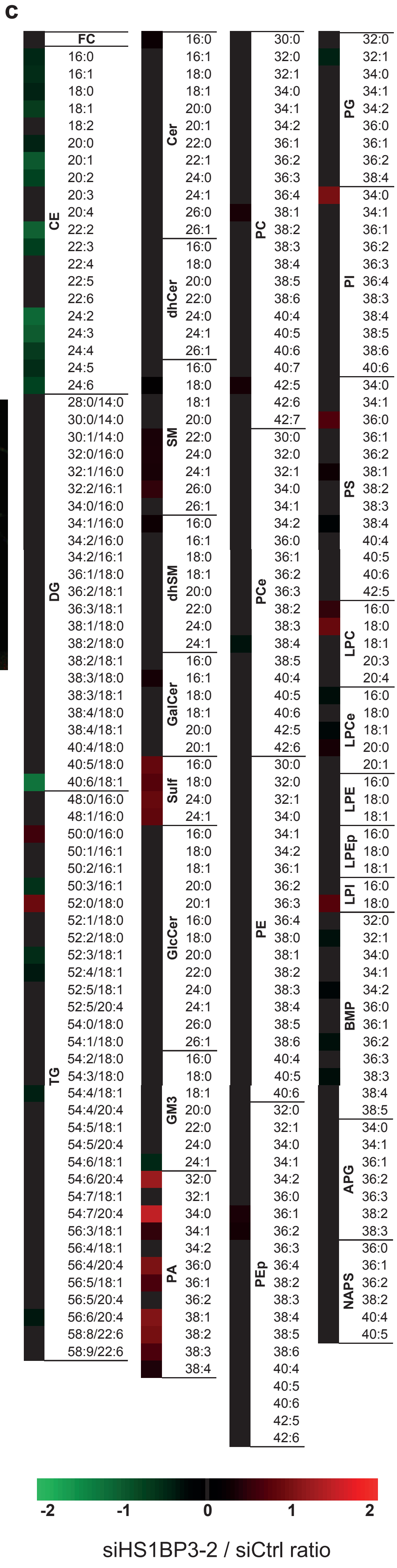

Supplementary Figure 4: HS1BP3 PX domain binds PA and affects several PA species, but not mTOR activity.

(**a**) Membranes spotted with the indicated lipids were incubated with 1 µg/mL of recombinant MBP-tagged proteins in a lipid protein overlay assay and bound proteins were detected with anti-MBP immunodetection. (**b**) HEK cells were transfected with GFP-HS1BP3-PX, starved, fixed and co-stained for endogenous ATG16L1. Yellow arrows indicate co-localization. Scale bar 10 µm. (**c**) HEK cells were treated with non-targeting or HS1BP3 siRNA. The total lipid content was extracted and analyzed by MS. Changes are indicated for all the analyzed lipid species by a heat-map with color indicating the relative fold-change due to HS1BP3 depletion. For a full list of all lipids with abbreviations, see Supplementary Table 1. (**d**) HEK GFP-LC3 cells were transfected with control siRNA or siRNA against HS1BP3 for 72 h and starved or not in the absence or presence of BafA1. Cell lysates were separated by SDS-PAGE and analyzed by immunoblotting. pAKT: phosphorylation of Ser473, p70-S6K: phosphorylation of Thr389. The data shown are representative of three independent experiments.

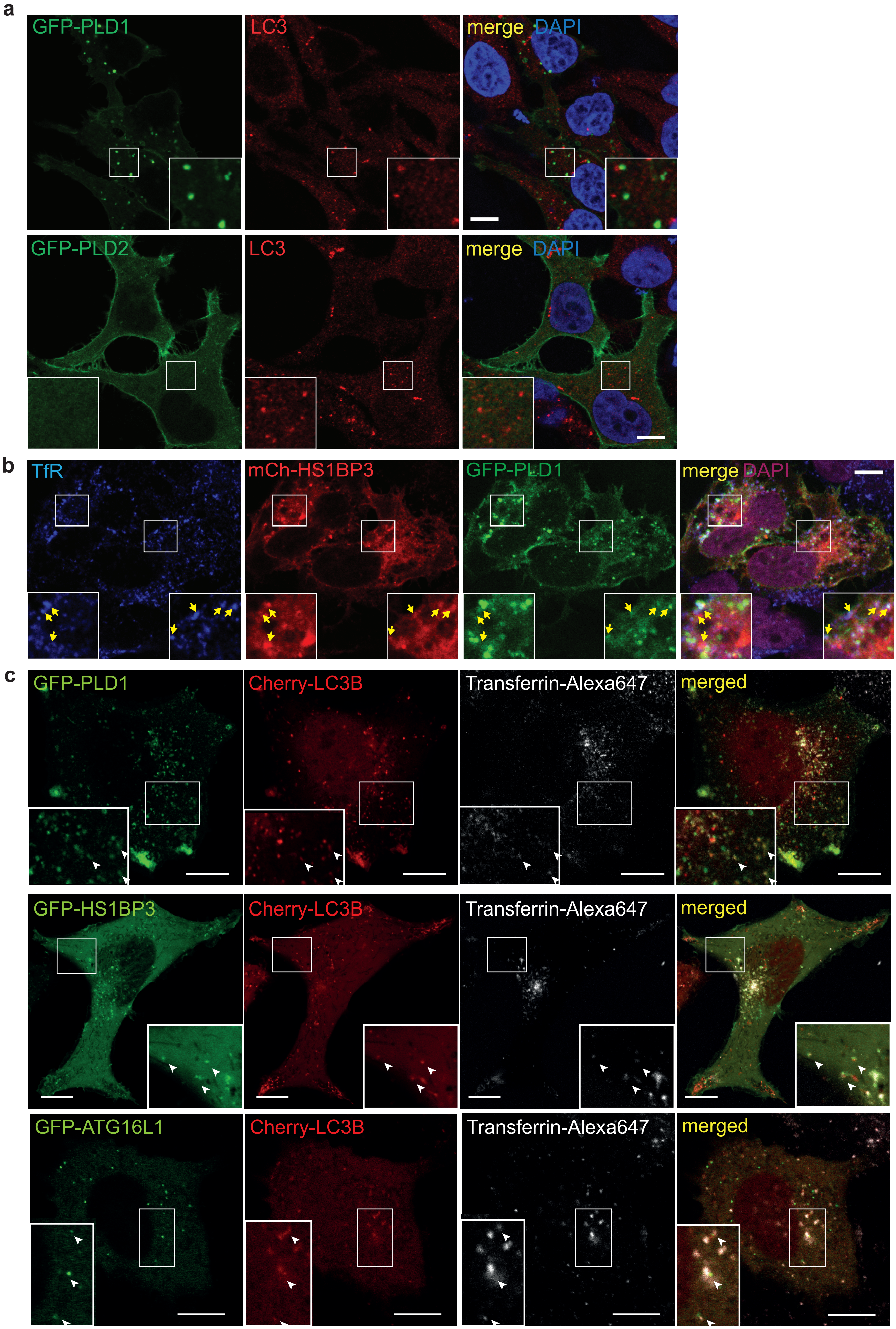

Supplementary Figure 5: HS1BP3 affects PLD1 on autophagy precursor membranes
(**a**) HEK cells were transfected to express GFP-tagged PLD1 or PLD2, starved and fixed then co-stained for endogenous LC3 and analyzed by confocal microscopy. (**b**) HEK cells were transfected to express GFP-tagged PLD1 and mCherry-HS1BP3, starved and fixed then co-stained for endogenous Transferrin receptor (TfR) and analyzed by confocal microscopy. All scale bars are 10 µm. (**c**) HEK293A cells expressing the indicated constructs were starved for 1h in EBSS containing 5µg/ml of transferrin-Alexa Fluor 647 conjugate and imaged live with Zeiss LSM710 confocal microscope. Shown are still image frames from live scan. Arrowheads point to structures positive for all three proteins. All scale bars are 10µm.

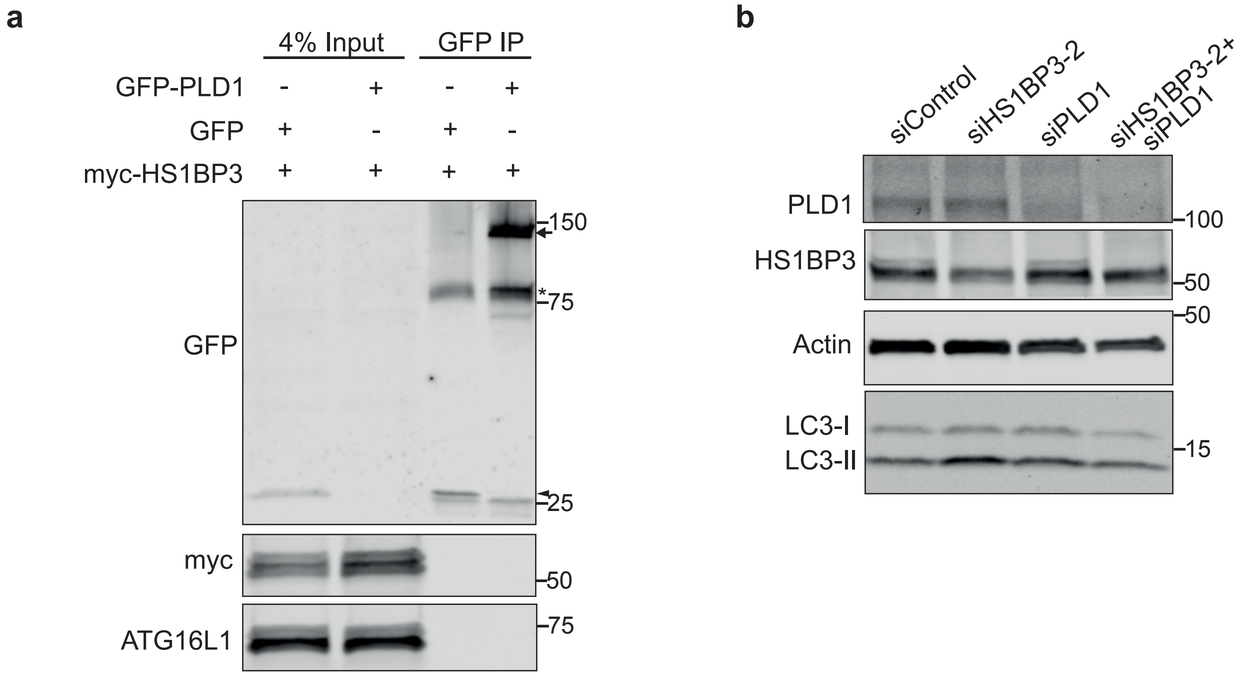

Supplementary Figure 6: HS1BP3 affects PLD1 on autophagy precursor membranes, but not through protein-protein interactions
(**a**) Immunoprecipitation of GFP or GFP-PLD1 from HEK cells transfected with GFP and myc-HS1BP3 or GFP-PLD1 and myc-HS1BP3. An interaction between GFP-PLD1 and myc-HS1BP3 or ATG16L1 was investigated by western blotting. On the GFP blot the arrow indicates GFP-PLD1, arrowhead GFP and the star an unspecific band. (**b**) HEK cells transfected with the indicated siRNA were starved then lysed and analyzed by western blotting with the indicated antibodies. The cells were treated in parallel with the ones analyzed by microscopy in Figure 7a.

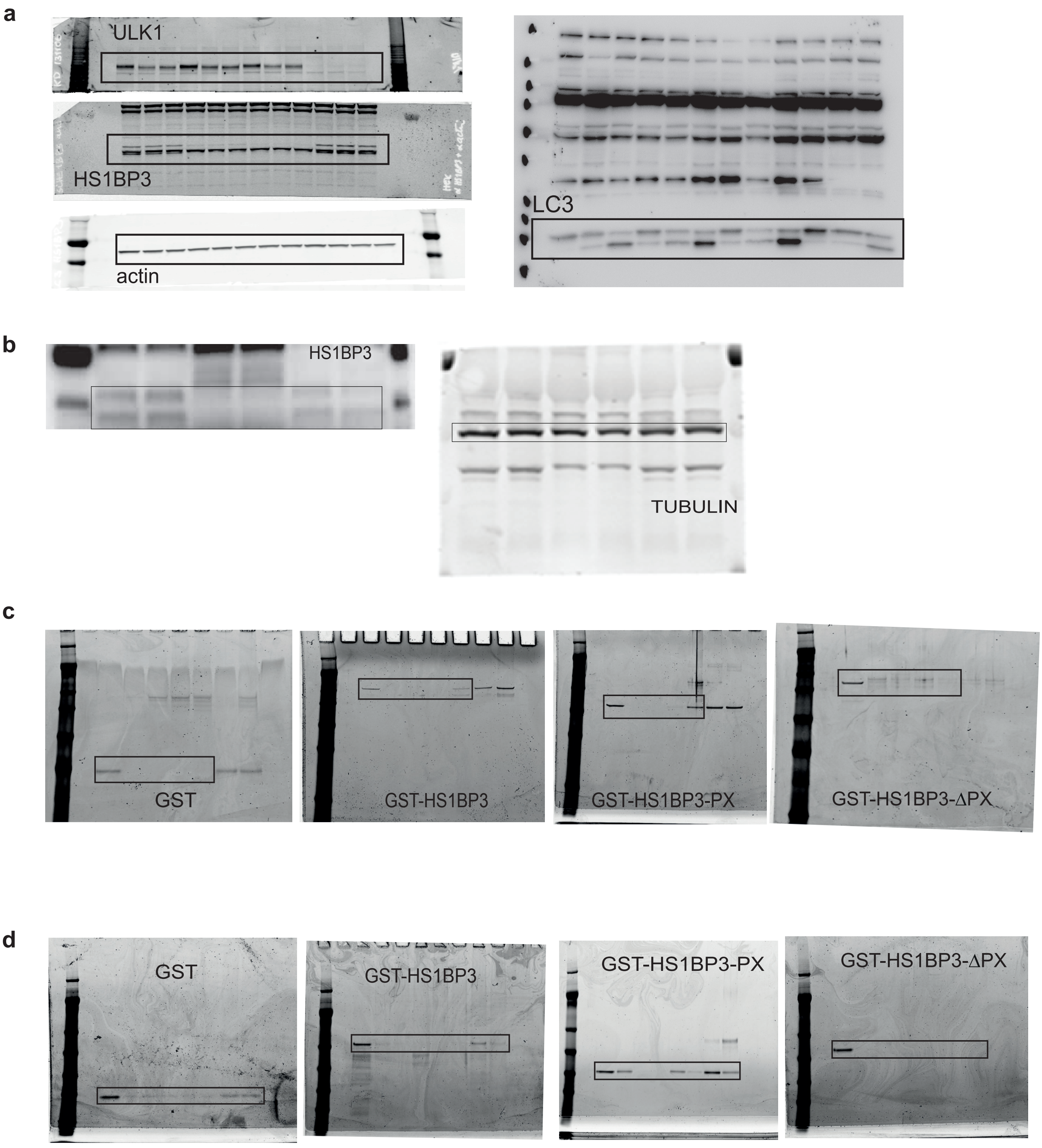

Supplementary Figure 7: Original scans used in the main text figures
(**a**) Blots from figure 1d
(**b**) Blots from figure 2c
(**c**) Gels from figure 4c
(**d**) Gels from figure 4d

Supplementary Table 1: Lipidomics data

**Lipid species**		**Average normalized mol %**		**SEM normalized mol %**		**t-test**
**Abbrev.**	**full name**	**siCtrl**	**siHS1 BP3-2**	**siCtrl**	**siHS1 BP3-2**	
FC	Free cholesterol	1	0.99	0.02	0.03	0.857
CE	Cholesteryl esters	1	0.80	0.03	0.05	0.007
DG	Diglycerides	1	1.00	0.17	0.17	0.995
TG	Triglycerides	1	1.10	0.04	0.10	0.391
Cer	Ceramide	1	1.12	0.03	0.06	0.112
dhCer	Dihydroceramide	1	1.00	0.07	0.07	0.967
SM	Sphingomyelin	1	1.16	0.04	0.03	0.017
dhSM	Dihydrosphingomyelin	1	1.15	0.03	0.07	0.069
GalCer	Galactosylceramide	1	1.08	0.03	0.07	0.287
Sulf	Sulfatide	1	1.80	0.21	0.14	0.010
GlcCer	Glucosylceramide	1	1.07	0.03	0.08	0.434
LacCer	Lactosylceramide	1	0.94	0.03	0.06	0.369
GM3	Monosialodihexosylganglioside	1	0.96	0.09	0.04	0.685
PA	Phosphatidic acid	1	1.95	0.14	0.26	0.010
PC	Phospatidylcholine	1	1.08	0.09	0.08	0.490
PCe	Phosphatidylcholine ether	1	0.88	0.07	0.05	0.219
PE	Phosphatidylethanolamine	1	1.05	0.07	0.07	0.583
PEp	Plasmalogen PE	1	1.11	0.06	0.04	0.154
PG	Phosphatidylglycerol	1	0.90	0.03	0.08	0.249
PI	Phosphatidylinositol	1	1.06	0.07	0.07	0.523
PS	Phosphatidylserine	1	1.07	0.04	0.07	0.401
LPC	Lysophosphatidylcholine	1	1.34	0.04	0.06	0.001
LPCe	Lysophosphatidylcholine ether	1	1.00	0.05	0.04	0.976
LPE	Lysophosphatidylethanolamine	1	1.09	0.03	0.11	0.456
LPEp	Plasmalogen LPE	1	1.23	0.06	0.17	0.225
LPI	Lysophosphatidylinositol	1	1.29	0.04	0.07	0.004
BMP	Bis(monoacylglycero)phosphates	1	0.85	0.01	0.04	0.005
APG	Acylphosphatidylglycerol	1	0.92	0.08	0.07	0.430
NAPS	N-acylphosphatidylserines	1	1.17	0.04	0.15	0.290

Supplementary Table 2: Plasmids used in this study.

Plasmid	Primers	Cloning
pEGFP-HS1BP3	5’-ATAGAATTCATGCAGTCCCCGGCGGTGCTC-3’ 5’-ATAGTCGACTCAGAAGAGGCTGGGGGCGG-3’	Into pEGFP.C2 using EcoRI and SalI restriction sites
pENTR-HS1BP3	5’-ATAGTCGACATGCAGTCCCCGGCGGTGCTC-3’ 5’-ATAGCGGCCGCTCAGAAGAGACTGGGGGCGG-3’	Amplification from cDNA library, into pENTR using SalI and NotI restriction sites
pENTR-HS1BP3-PX	5’-ATAGTCGACATGCAGTCCCCGGCGGTGCTC-3’ 5’ATAGCGGCCGCTCAGGATCTGGTACCTAAGAACTC-3’	Amplification from pENTR-HS1BP3, into pENTR using SalI and NotI restriction sites
pENTR-HS1BP3-ΔPX	5’-ATAGTCGACGCTGCAGGGCTCACCAGCAG-3’ 5’-ATAGCGGCCGCTCAGAAGAGACTGGGGGCGG-3’,	Amplification from pENTR-HS1BP3, into pENTR using SalI and NotI restriction sites
pTH1-HS1BP3 (MBP tag)	-	Gateway LR cloning from pENTR-HS1BP3
pTH1-HS1BP3-PX (MBP tag)	-	Gateway LR cloning from pENTR HS1BP3-PX
pTH1-HS1BP3-ΔPX (MBP tag)	-	Gateway LR cloning from pENTR HS1BP3-ΔPX
pDEST-mCherry-HS1BP3	-	Gateway LR cloning from pENTR-HS1BP3
pTH1-2xFYVE-Hrs (MBP tag)	-	Gateway LR cloning from pENTR-2xFYVE-Hrs
pGEX-2xFYVE-Hrs	-	[1]
pCI2Flag Cortactin	-	Kindly provided by J. K. Burkhardt
pGEX kG hHS1 SH3	-	Kindly provided by J. K. Burkhardt
pGEX kG hHS1 SH3 W->Y	-	Kindly provided by J. K. Burkhardt
pGEX kG hcortactin SH3	-	Kindly provided by J. K. Burkhardt
pGEX kG h cortactin SH3 W->Y	-	Kindly provided by J. K. Burkhardt
pCGN-HA-hPLD1b	-	Kindly provided by M. Frohman [2]
pEGFP-C1-hPLD1b	-	Kindly provided by M. Frohman [2]
pEGFP-C1-mPLD2	-	Kindly provided by M. Frohman [2]
pDEST15-LC3B	-	[3]

(GST tag)		
pDEST15-GABARAP (GST tag)	-	[3]
pDEST15-HS1BP3-PX (GST tag)	-	Gateway LR cloning from pENTR HS1BP3-PX
pDEST53-p62 (in vitro translation)	-	[3]
pDEST53-HS1BP3 (in vitro translation)	-	Gateway LR cloning from pENTR-HS1BP3
pSP64 Poly (A)-Hs-Hs1bp3	5’ taaaAAGCTTATGCAGTCCCCGGCGGTG 3’<br>5’ taaaGAGCTCTCAGAAGAGGCTGGGGGC 3’	Expression of HS1BP3 in zebrafish
pEGFP-C2 DFCP1	-	Kindly provided by Nicholas Ktistakis
pEGFP – p62	-	-
pDEST-EGFP-LC3B	-	-
pEGFP(C1)-Atg16L	-	-
pEGFP-Atg14L	-	Kindly provided by Tamotsu Yoshimori

Supplementary references

1. Gillooly, D.J. *et al.* Localization of phosphatidylinositol 3-phosphate in yeast and mammalian cells. *The EMBO journal* **19**, 4577-4588 (2000).
2. Hammond, S.M. *et al.* Human ADP-ribosylation factor-activated phosphatidylcholine-specific phospholipase D defines a new and highly conserved gene family. *J Biol Chem* **270**, 29640-29643 (1995).
3. Pankiv, S. *et al.* p62/SQSTM1 binds directly to Atg8/LC3 to facilitate degradation of ubiquitinated protein aggregates by autophagy. *J.Biol.Chem.* **282**, 24131-24145 (2007).
